# Cardiac “hypertrophy” phenotyping: differentiating aetiologies with increased left ventricular wall thickness on echocardiography

**DOI:** 10.3389/fcvm.2023.1183485

**Published:** 2023-07-03

**Authors:** Aaisha Ferkh, Catherina Tjahjadi, Luke Stefani, Paul Geenty, Karen Byth, Kasun De Silva, Anita C. Boyd, David Richards, Peter Mollee, Dariusz Korczyk, Mark S. Taylor, Fiona Kwok, Eddy Kizana, Arnold C. T. Ng, Liza Thomas

**Affiliations:** ^1^ Westmead Clinical School, University of Sydney, Westmead, NSW, Australia; ^2^ Cardiology Department, Westmead Hospital, Westmead, NSW, Australia; ^3^ Cardiology Department, Princess Alexandra Hospital, Brisbane, QLD, Australia; ^4^ WSLHD Research and Education Network, Westmead Hospital, Westmead, NSW, Australia; ^5^ Westmead Private Cardiology, Westmead, NSW, Australia; ^6^ Haematology Department, Princess Alexandra Hospital, Brisbane, QLD, Australia; ^7^ School of Medicine, University of Queensland, Brisbane, QLD, Australia; ^8^ Department of Clinical Immunology and Allergy, Westmead Hospital, Westmead, NSW, Australia; ^9^ Haematology Department, Westmead Hospital, Westmead, NSW, Australia; ^10^ Centre for Heart Research, The Westmead Institute for Medical Research, Westmead, NSW, Australia; ^11^ South-West Clinical School, University of New South Wales, Liverpool, NSW, Australia

**Keywords:** cardiac amyloidosis, Fabry disease, cardiac hypertrophy, infiltrative cardiomyopathies, echocardiography, strain imaging

## Abstract

**Aims:**

Differentiating phenotypes of cardiac “hypertrophy” characterised by increased wall thickness on echocardiography is essential for management and prognostication. Transthoracic echocardiography is the most commonly used screening test for this purpose. We sought to identify echocardiographic markers that distinguish infiltrative and storage disorders that present with increased left ventricular (LV) wall thickness, namely, cardiac amyloidosis (CA) and Anderson–Fabry disease (AFD), from hypertensive heart disease (HHT).

**Methods:**

Patients were retrospectively recruited from Westmead Hospital, Sydney, and Princess Alexandra Hospital, Brisbane. LV structural, systolic, and diastolic function parameters, as well as global (LVGLS) and segmental longitudinal strains, were assessed. Previously reported echocardiographic parameters including relative apical sparing ratio (RAS), LV ejection fraction-to-strain ratio (EFSR), mass-to-strain ratio (MSR) and amyloidosis index (AMYLI) score (relative wall thickness × *E*/*e*′) were evaluated.

**Results:**

A total of 209 patients {120 CA [58 transthyretin amyloidosis (ATTR) and 62 light-chain (AL) amyloidosis], 31 AFD and 58 HHT patients; mean age 64.1 ± 13.7 years, 75% male} comprised the study cohort. Echocardiographic measurements differed across the three groups, The LV mass index was higher in both CA {median 126.6 [interquartile range (IQR) 106.4–157.9 g/m^2^]} and AFD [median 134 (IQR 108.8–152.2 g/m^2^)] vs. HHT [median 92.7 (IQR 79.6–102.3 g/m^2^), *p* < 0.05]. LVGLS was lowest in CA [median 12.29 (IQR 10.33–15.56%)] followed by AFD [median 16.92 (IQR 14.14–18.78%)] then HHT [median 18.56 (IQR 17.51–19.97%), *p* < 0.05]. Diastolic function measurements including average *e*′ and *E*/*e*′ were most impaired in CA and least impaired in AFD. Indexed left atrial volume was highest in CA. EFSR and MSR differentiated secondary (CA + AFD) from HHT [receiver operating curve–area under the curve (ROC-AUC) of 0.80 and 0.91, respectively]. RAS and AMYLI score differentiated CA from AFD (ROC-AUC of 0.79 and 0.80, respectively). A linear discriminant analysis with stepwise variable selection using linear combinations of LV mass index, average *e*′, LVGLS and basal strain correctly classified 79% of all cases.

**Conclusion:**

Simple echocardiographic parameters differentiate between different “hypertrophic” cardiac phenotypes. These have potential utility as a screening tool to guide further confirmatory testing.

## Introduction

Echocardiographic cardiac “hypertrophy” [defined as increased left ventricular (LV) wall thickness on echocardiography] is a hallmark of infiltrative cardiomyopathies such as cardiac amyloidosis (CA), lysosomal storage diseases such as Anderson–Fabry disease (AFD), and conditions with true myocyte hypertrophy such as hypertrophic cardiomyopathy (HCM) and hypertensive heart disease (HHT). Patients with infiltrative/storage aetiologies of cardiac “hypertrophy” often experience a delay in diagnosis (1), with significant implications for management, as disease-specific therapies are now available. Endomyocardial biopsy is the gold standard for diagnosis but is invasive and often non-diagnostic (2). Whilst non-invasive imaging strategies include bone scintigraphy for diagnosing transthyretin (ATTR) CA (3) and cardiac magnetic resonance imaging (CMR) for CA and AFD (4), echocardiography is the initial, widely available and relatively inexpensive modality for screening, offering an attractive alternative to identify aetiology in cardiomyopathies with a “hypertrophic” phenotype.

Previous studies have investigated novel echocardiographic parameters. Phelan et al. (5) demonstrated that relative apical sparing ratio (RAS—ratio of apical to mid + basal longitudinal strain) >1 was sensitive and specific for CA, vs. patients with HCM and aortic stenosis. Left ventricular ejection fraction (LVEF)-to-strain ratio (EFSR) was a strong discriminator between CA and HCM (6). More recently, the increased wall thickness (IWT) score distinguished CA from other causes of LV “hypertrophy”, including HCM (7). An amyloidosis index (AMYLI) score [relative wall thickness (RWT) × *E*/*E*′] <2.2 was sensitive, excluding CA (8). Finally, our group (9) demonstrated that the LV mass-to-strain ratio (MSR) accurately distinguished ATTR and light-chain (AL) amyloidosis.

However, most of the above reports include small patient populations and involve complex multiparametric measures (e.g., IWT score), and none have included patients with AFD. The studies above have extensively investigated CA vs. HCM, and reports do not focus on differentiating CA from AFD. Our primary aim was to investigate echocardiographic parameters (including EFSR, MSR, AMYLI and RAS) that distinguish secondary causes (infiltrative and storage) that present with increased LV wall thickness including CA and AFD from HHT.

## Materials and methods

### Patient selection

Consecutive CA patients were retrospectively recruited from amyloidosis clinics at Westmead Hospital, Sydney (2009–2021), and Princess Alexandra Hospital, Brisbane (2013–2021), both tertiary state referral centres for amyloidosis. The inclusion criteria include (1) a positive cardiac biopsy for amyloidosis, (2) positive bone scintigraphy for ATTR amyloidosis (grade ≥2 cardiac tracer uptake) and absence of monoclonal gammopathy of uncertain significance (10) or (3) in the case of AL, a biopsy showing AL amyloid deposits with an unexplained increased wall thickness and elevated N-terminal prohormone of brain natriuretic peptide (NT-proBNP) (11). AFD patients were consecutively recruited from the Genetic Medicine Department at Westmead Hospital (2001–2020), and all had positive AFD gene tests. HHT patients were selected from a departmental database (2010–2021). Only patients with mean LV wall thickness ≥11 mm were included in all groups to ensure a “hypertrophic” phenotype. Patients with other structural heart diseases or major valvulopathy were excluded. The ethics committees in both participating institutions approved the study.

### Echocardiographic measurements

A comprehensive transthoracic echocardiogram, including 2D, colour, and Doppler, was performed in all patients in accordance with the American Society of Echocardiography/European Association of Cardiovascular Imaging guidelines (12), using General Electric (GE) EchoPAC version 204 (Milwaukee, WI, United States). Experienced researchers with extensive training in echocardiographic and strain measurements performed the measurements.

In the parasternal long-axis view, LV interventricular septum and posterior wall thickness and LV end-diastolic diameter (LVEDD) were measured in end-diastole. Mean wall thickness (MWT) was the average of LV interventricular septum and posterior wall thickness. RWT was calculated as 2 × posterior wall thickness/LVEDD. LV ejection fraction (LVEF) was calculated using Simpson's biplane-derived LV end-diastolic and LV end-systolic volumes. The LV mass index (LVMI) was derived using the Devereux formula and indexed to the body surface area (BSA). Left atrial volume (LAVI) was obtained from the four- and two-chamber apical views using the area-length method and indexed to BSA. Mitral inflow *E* and *A* velocities and *E*/*A* ratio were obtained for LV diastolic function. Tissue Doppler septal and lateral annular *e*′ velocities were obtained. Average *e*′ and *E*/average *e*′ were calculated. Tricuspid annular plane systolic excursion (TAPSE) was measured for right ventricular (RV) function.

Two-dimensional speckle tracking strain analysis (GE EchoPAC software version 204 Q-Analysis) was utilised to measure LV global longitudinal strain (LVGLS) and RV free-wall strain (RVFWS). Frame rates of >60 frames per second and an average of measurements from three cardiac cycles were used; in atrial fibrillation (AF), an average of three cycles with similar R-R intervals was utilised. LVGLS was measured from the apical 4-chamber, 2-chamber, and long-axis views by tracing the endocardium and manually adjusting the region of interest to myocardial thickness, providing an 18-segment LV model (6 segments per apical view). LVGLS was the average of 18 segments. Basal, mid, and apical segmental strains were calculated as the average of six segments at each level. RVFWS was the average of the three free-wall segments. Absolute values for strains are reported.

**Figure 1:**
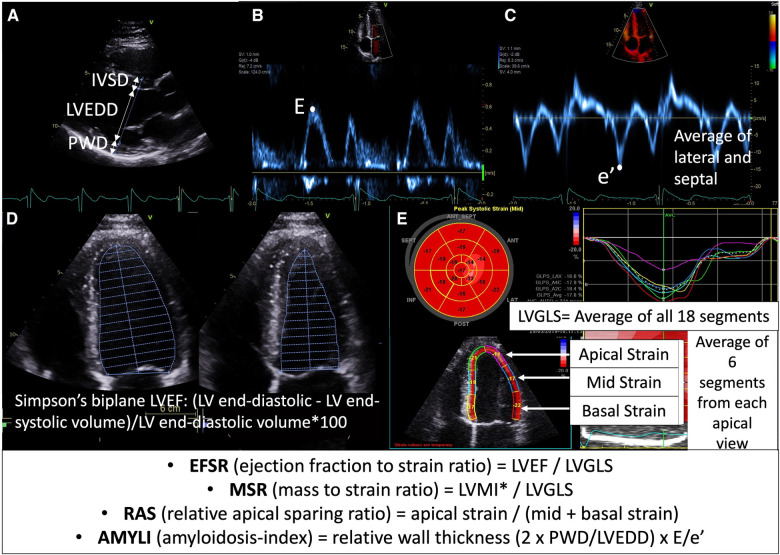
Calculation of echocardiographic formulae evaluated in this study: (**A**) parasternal long-axis view demonstrating wall thickness (IVSD, interventricular septal diameter; PWD, posterior wall diameter) and cavity size (LVEDD) measurement, (**B**) mitral valve inflow Doppler demonstrating *E* velocity, (**C**) tissue Doppler of mitral valve demonstrating *e*′ velocity (average of septal and lateral used in *E*/*e*′ formula), (**D**) Simpson's LVEF measurement (apical four-chamber view demonstrated here, but biplane four- and two-chamber views are used in calculation); (**E**) longitudinal strain measurement demonstrating segmental strain (average of six segments across apical four-chamber, two-chamber and long-axis views at each of basal, mid and apical levels) and global longitudinal strain (LVGLS—average of all 18 segments). *LVMI was calculated using the Devereux formula (i.e., 0.8 {1.04 [(LVEDD + IVSD + PWD)^3^ − LVEDD^3^]} + 0.6) and indexed to body surface area.

*E*/*A* could only be derived in sinus rhythm (SR) (*n* = 96). In the older AFD studies, the RV-S′ velocity was often not measured and hence was excluded from the results. TAPSE could only be measured in 19/31, and RVFWS in 15. Parameter ratios mentioned previously such as EFSR (LVEF:LVGLS), RAS (apical strain/mid + basal strain), AMYLI (RWT × *E*/*e*′) and MSR (LVMI:LVGLS) were derived and evaluated (Figure 1).

### Statistical analysis

IBM SPSS Statistics version 28 (SPSS, Chicago, IL, United States) was used to analyse data. Categorical variables were summarised using frequencies and percentages. Median and interquartile range (IQR) were used for continuous variables due to their skewed distributions. Chi-squared tests were used to test the association between categorical variables. The Mann–Whitney test and Kruskal–Wallis nonparametric analysis of variance examined differences for continuous variables. Two-tailed tests with a 5% significance level were used throughout.

The receiver operating curve–area under the curve (ROC-AUC) was used to quantify the performance of continuous variables in differentiating (1) secondary (infiltrative/storage) aetiology (CA and AFD) vs. HHT and (2) CA vs. AFD. To compare infiltrative/storage causes (CA and AFD) with HHT, a cut-point achieving high sensitivity and reasonable specificity was selected for screening purposes. For CA vs. AFD, a cut-point that evenly balanced specificity and sensitivity was set. A simple two-step tree-based classifier was used to classify patients into one of three groups, and its performance was evaluated. Since age differed significantly between AFD and the other groups, a general linear model was used to estimate age-adjusted differences in the four parameters (EFSR, MSR, RAS and AMYLI) between secondary causes (infiltrative/storage; CA + AFD) and HHT groups and between CA and AFD.

The potential for developing machine learning classifiers based solely on candidate variables easily measured from echocardiograms was explored. As proof of concept, a simple supervised learning classifier, linear discriminant analysis (LDA) (13–15) with stepwise variable selection, was used to estimate linear combinations of candidate variables, which best explained differences between CA, AFD and HHT. Leave-one-out cross-validation was used to estimate the diagnostic performance of LDA. A scatterplot of the second vs. first canonical discriminant function illustrates the separation of the groups. A separate LDA was performed in a subgroup of patients with mild–moderately increased wall thickness (MWT <16 mm).

Intraobserver and interobserver variability was performed for LV mass and strain measurements. Intraclass correlation coefficients were above 0.9 for all measurements demonstrating good reproducibility.

## Results

A total of 120 CA patients (62 AL and 58 ATTR), 31 AFD and 58 HHT patients comprised the study group. Table 1 presents clinical and echocardiographic parameters. All three groups had a male predominance, with no difference in sex distribution across groups. Patients with AFD were younger than those of other groups. HHT patients had higher blood pressure and body mass index (BMI). All AFD and HHT patients were in SR. A total of 23 CA patients were in AF/flutter and 1 in paced rhythm.

**Table 1:** Baseline characteristics and echocardiographic parameters in CA, AFD, and HHT patient groups.

	CA (*n* = 120)	AFD (*n* = 31)	HHT (*n* = 58)	Total (*n* = 209)	*p* value*
Sex (male)	95 (79.2)	20 (64.5)	42 (72.4)	157 (75.1%)	0.208
Age (years)	69 (60–76)^a^	46 (40–54)^b^	72 (63–76)	66 (55–74)	<0.001
SBP (mm Hg)	125 (110–135)	125 (112–130)^b^	140 (130–150)^c^	130 (119–140)	<0.001
DBP (mm Hg)	73 (68–80)	76 (70–80)^b^	80 (75–86)^c^	77 (70–80)	<0.001
BMI (kg/m^2^)	26.4 (23.9–29.7)	24.7 (22.4–28.9)^b^	30.5 (27.4–35.3)^c^	27.1 (24.2–31)	<0.001
HR (bpm)	73 (60–83)^a^	63 (60–72)^b^	74 (67–80)	71.5 (61–81)	0.035
LVEF biplane (%)	53 (49–59)^a^	61.3 (57.3–65.2)	60 (58–63)^c^	58 (51–62)	<0.001
LVEDD (mm)	43 (39–47.5)^a^	47 (43.7–51)^b^	41 (38–45)^c^	43 (39–48)	<0.001
MWT (mm)	15 (13–16)^a^	13 (12–15)^b^	12 (12–13)^c^	13 (12–16)	<0.001
LVMI (g/m^2^)	126.6 (106.4–157.9)	134 (108.8–152.2)^b^	92.7 (79.6–102.3)^c^	114.1 (97.9–144.8)	<0.001
RWT (mm)	0.65 (0.52–0.77)^a^	0.49 (0.45–0.63)	0.58 (0.52–0.67)^c^	0.6 (0.51–0.72)	<0.001
*E*/*A* (m/s)	1.09 (0.8–1.85)	1.23 (1.1–1.5)^b^	0.79 (0.69–0.97)^c^	0.99 (0.75–1.49)	<0.001
Average *e*′ (cm/s)	5 (4–6)^a^	7.7 (6.5–9.2)^b^	6 (5.5–7.5)^c^	6 (4.5–7)	<0.001
*E*/*e*′ (cm/s)	15.63 (11.64–20.57)^a^	9.77 (8.66–12.87)	10.4 (8.63–12.67)^c^	12.88 (9.71–17.17)	<0.001
LAVI (ml/m^2^)	49.79 (39.01–59)^a^	36.91 (26.42–42.09)	31.99 (26.83–38.09)^c^	40.31 (31.96–52.41)	<0.001
TAPSE (mm)	18.6 (15.2–21.1)^a^	22 (20–26)	21 (20–24)^c^	20 (17–23)	<0.001
LVGLS (%)	12.29 (10.33–15.56)^a^	16.92 (14.14–18.78)^b^	18.56 (17.51–19.97)^c^	15.7 (11.54–18.36)	<0.001
Basal strain (%)	9.09 (5.5–11.4)^a^	14.09 (12.67–16.24)^b^	16.3 (14.76–18.53)^c^	12.3 (7.5–15.75)	<0.001
Mid strain (%)	11.82 (9.6–14.94)^a^	16.26 (13.71–18.12)^b^	18.2 (16.5–20.4)^c^	15.2 (10.8–17.84)	<0.001
Apical strain (%)	17.4 (14.9–19.9)^a^	19.55 (16.84–22.53)^b^	22.39 (20.05–24.08)^c^	19.33 (16.18–22.58)	<0.001
RVFWS (%)	19.5 (15.4–23.78)^a^	24.45 (21.93–25.8)	22.71 (21.21–24.85)^c^	21.42 (17.2–24.46)	<0.001
EFSR	4.09 (3.5–4.99)	3.64 (3.32–4.25)^b^	3.24 (3.05–3.48)^c^	3.62 (3.2–4.5)	<0.001
MSR	10.19 (7.56–15.06)^a^	8.19 (5.99–11.06)^b^	4.95 (4.31–5.56)^c^	7.85 (5.31–11.71)	<0.001
RAS	0.851 (0.702–1.084)^a^	0.64 (0.541–0.745)	0.645 (0.561–0.709)^c^	0.739 (0.614–0.907)	<0.001
AMYLI	9.64 (6.87–15.76)^a^	5.74 (4.35–7.1)	5.91 (5.03–7.71)^c^	7.71 (5.49–11.82)	<0.001

AFD, Anderson–Fabry disease; AMYLI score, RWT×*E*/*e*′; BMI, body mass index; CA, cardiac amyloid; *E*/*A*, mitral inflow *E*/*A* velocity ratio; *e*′, mitral valve tissue Doppler *e*′ velocity; EFSR, LVEF-to-strain ratio; HHT, hypertensive heart disease; LAVI, left atrial volume index; LVEDD, LV end-diastolic diameter; LVEF, left ventricular ejection fraction; LVGLS, left ventricular global longitudinal strain; LVMI, left ventricular mass index; MSR, mass-to-strain ratio; MWT, mean wall thickness; RAS, relative apical sparing ratio; RVFWS, right ventricular free-wall strain; RWT, relative wall thickness; TAPSE, tricuspid annular plane systolic excursion; SBP, Systolic blood pressure; DBP, Diastolic blood pressure; HR, Heart rate.

Data presented as median (lower quartile–upper quartile) for continuous variables and frequency (percentage) for categorical variables.

* *p* value for sex derived using Chi-squared test, for RV-S′ (unavailable in AFD) using Mann–Whitney test and for remaining variables using Kruskal–Wallis test.

^a^ CA vs. AFD (*p* < 0.05).

^b^ AFD vs. HHT (*p* < 0.05).

^c^ CA vs. HHT (*p* < 0.05).

With echocardiographic parameters, LVEF was lower in CA compared with both AFD and HHT, although the median LVEF for CA was low–normal (53%). LVMI was higher in both CA and AFD vs. HHT. MWT was highest in CA, whilst LVEDD was highest in AFD. Doppler measurements including average *e*′ and *E*/*e*′ were most impaired in CA and least impaired in AFD. LAVI was highest in CA. RV function measures (TAPSE, RVFWS) were lowest in CA and similar/preserved in AFD and HHT. LVGLS was lowest in CA followed by AFD, then HHT. This trend was also observed for segmental strain, predominantly basal strain, and RAS was highest in CA.

**Figure 2:**
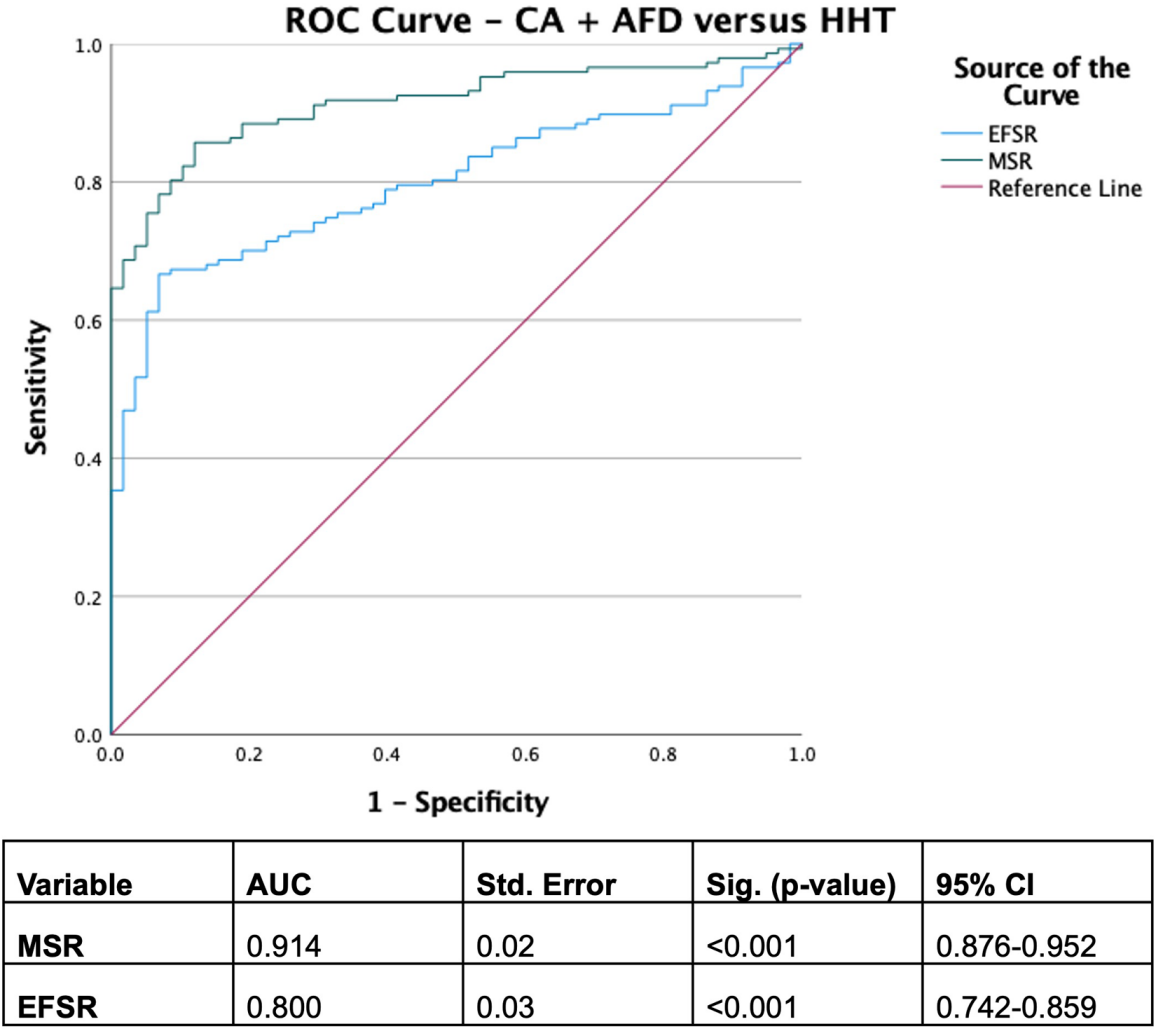
ROC curve for secondary infiltrative and storage aetiologies (cardiac amyloidosis and Anderson–Fabry disease) vs. hypertensive cardiomyopathy.

**Figure 3:**
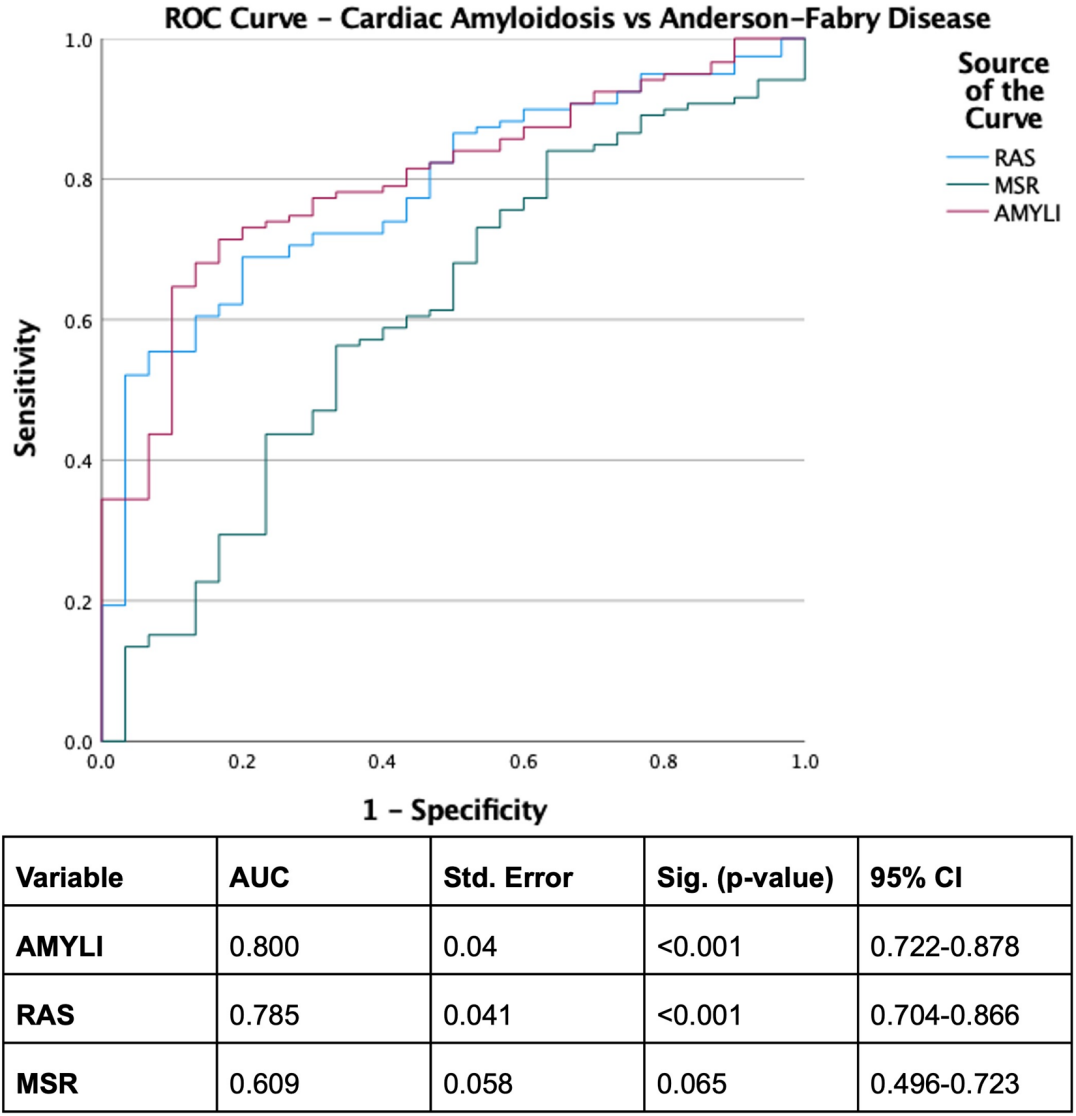
ROC curve for differentiating cardiac amyloidosis from Anderson–Fabry disease. AMYLI score, relative wall thickness × *E*/*e*′.

With derived parameters, EFSR was similar, whilst MSR differentiated CA and AFD (*p* = 0.058 and *p* = 0.044, respectively). Both EFSR and MSR differentiated the secondary causes (CA and AFD) vs. HHT (*p* < 0.001). RAS and AMYLI were comparable between AFD and HHT (0.530 and 0.379, respectively) but differentiated CA vs. AFD and HHT (*p* < 0.001 for both). The ROC-AUC of EFSR and MSR for secondary causes (CA and AFD) vs. HHT was 0.80 for EFSR and 0.91 for MSR, respectively (Figure 2). The sensitivity and specificity of EFSR ≥3.3 for detecting secondary causes (CA + AFD vs. HHT) were 80% (95% CI 72%–86%) and 59% (95% CI 45%–71%), respectively, and corresponding values for MSR ≥5.3 were 91% (95% CI 86%–95%) and 71% (95% CI 57%–82%). The ROC-AUC for MSR, RAS and AMYLI for CA vs. AFD were 0.79, 0.80 and 0.61, respectively (Figure 3). The sensitivity and specificity of RAS ≥0.74 (74%) for detecting CA (vs. AFD) were 71% (95% CI 62%–79%) and 74% (95% CI 55%–88%), and corresponding values for AMYLI ≥7.6 were 71% (95% CI 62%–79%) and 83% (95% CI 65%–94%).

A two-step approach of MSR ≥5.3 and AMYLI ≥7.6 to differentiate CA, AFD and HHT was assessed. The predicted group for each case was determined by assignment to “predicted HHT” if MSR <5.3, “predicted CA” if MSR ≥5.3 and AMYLI ≥7.6 and “predicted AFD” if MSR ≥5.3 and AMYLI <7.6. Cross-tabulation compared the true group with the predicted group and accurately predicted 70% of all cases (69% CA, 77% AFD and 69% HHT).

After age adjustment, EFSR and MSR still differentiated secondary causes (CA + AFD) from HHT (*p* < 0.001 for both), and RAS and AMYLI differentiated CA from AFD (*p* < 0.05 for both) (Supplementary data).

**Figure 4:**
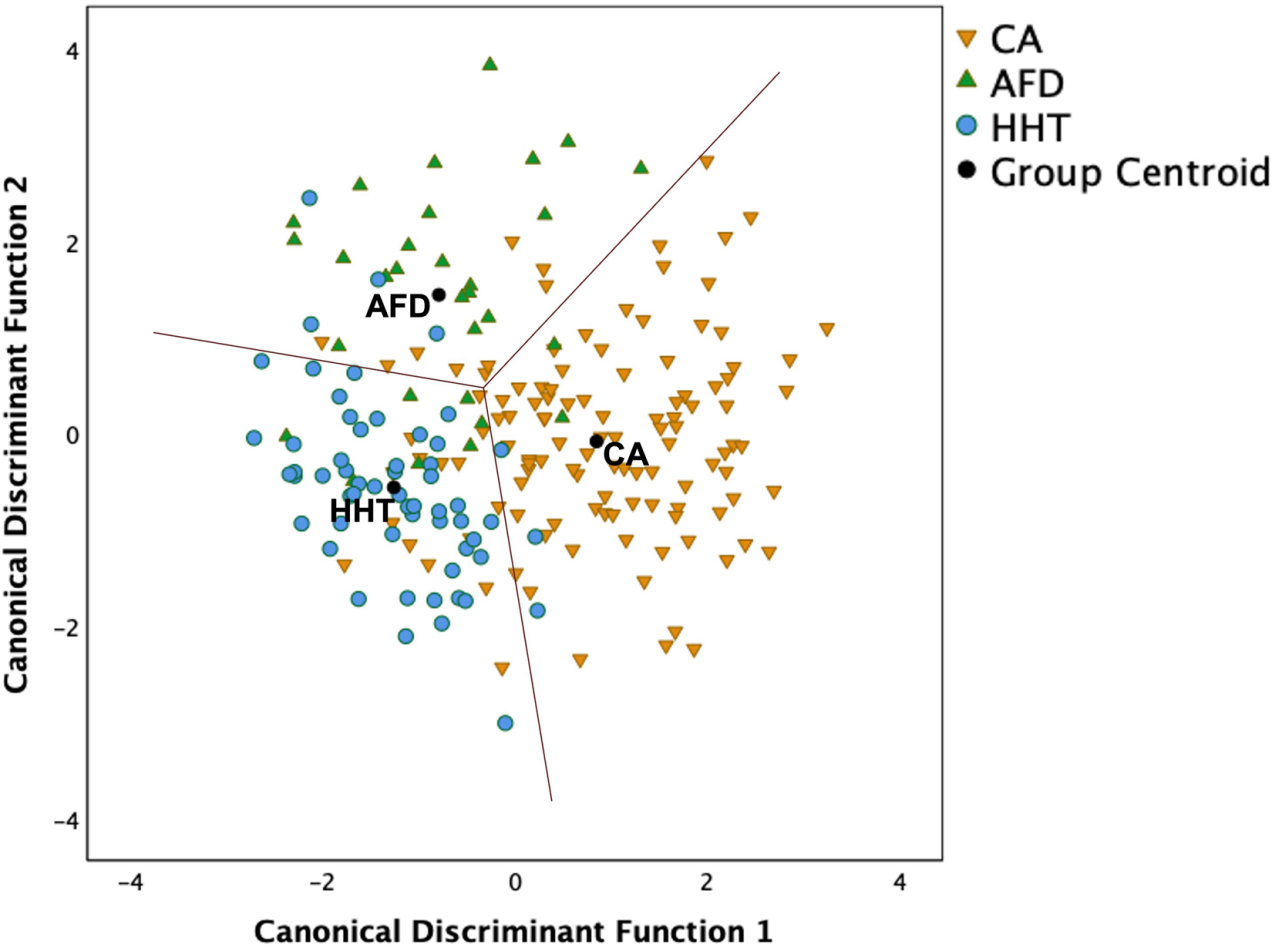
Scatterplot of two canonical discriminant functions (linear combinations of left ventricular mass index, average *e*′, left ventricular global longitudinal strain and basal strain) derived using linear discriminant analysis to differentiate the three groups. Overall, 78.6% of cases were correctly classified using leave-one-out cross-validation, comprising 78% CA, 70% AFD, and 85% HHT.

Figure 4 illustrates the LDA results based solely on candidate variables easily measured from echocardiograms. The two canonical discriminant functions that best explained the differences between the three groups were linear combinations of LVMI, average *e*′, LVGLS and basal strain (Supplementary data). Leave-one-out cross-validation estimated that 79% of all cases were correctly classified (i.e., 78% CA, 70% AFD and 85% HHT), a 9% improvement on the two-step approach using MSR ≥5.3 status and AMYLI ≥7.6 status. These findings need validation in independent samples of patients.

In the subgroup of patients with mild–moderately increased wall thickness (MWT <16 mm; 72 CA, 24 AFD and 58 HHT patients), RAS and AMYLI differentiated CA from AFD (*p* < 0.001) whilst EFSR and MSR differentiated AFD from HHT (*p* = 0.008 and *p* < 0.001, respectively) (Supplementary data). For secondary causes (CA + AFD) vs. HHT, the ROC-AUC was 0.72 for EFSR and 0.87 for MSR (Supplementary Figure S1). For CA vs. AFD, the ROC-AUC was 0.75 for RAS and 0.79 for AMYLI (Supplementary Figure S2). LDA of this subgroup (Supplementary Figure S3) demonstrated basal strain, LVMI and average *e*′ as candidates for inclusion in the final model. The model correctly predicted 79% of all subgroups on cross-validation (75% CA, 83% AFD and 83% HHT).

## Discussion

This study has several key findings. Firstly, we demonstrate that simple echocardiographic metrics differentiate “hypertrophic” phenotypes. MSR discriminated secondary (infiltrative and storage) causes (CA + AFD) from HHT (ROC-AUC 0.91; MSR ≥5.3 with 91% sensitivity, 71% specificity for CA + AFD), whilst the AMYLI score discriminated CA from AFD (ROC-AUC 0.80, with AMYLI ≥7.6 having 71% sensitivity and 83% specificity for CA). Secondly, we demonstrated that an LDA based on four echocardiographic measurements, i.e., basal strain, LVGLS, LVMI, and average *e*′, has a good discriminative ability. It accurately predicted the correct group for 79% of cases overall. A similar result for LDA was seen in the subset of early disease patients with mild–moderately increased wall thickness.

### Differing clinical and echocardiographic characteristics of CA, AFD and HHT

AFD patients were younger as this hereditary condition manifests earlier in life (16), whilst CA, particularly ATTR, occurs predominantly in the elderly (17). The male predominance of these conditions is recognised, particularly ATTR-CA, and AFD, which is X-linked (16, 18). However, the inherent underrepresentation of females in all cohorts may also be due to delays in diagnosis (19). BMI was higher in HHT, likely due to the relationship between obesity and hypertension (20).

With respect to echocardiographic parameters, LVMI was highest in CA and AFD. LV cavity size was higher in AFD compared with CA, and consequently, RWT was lowest in AFD. Small LV cavity size is a hallmark of advanced CA (21), but to our knowledge, there have been no reports of comparatively preserved LV cavities in AFD patients. The relatively preserved diastolic function markers (average *e*′ and *E*/*e*′) seen in AFD patients could partly be attributed to their younger age. Similarly, previous AFD studies have reported mild diastolic dysfunction, with only a small percentage of AFD patients demonstrating restrictive filling (22, 23). Left atrial volume was highest in CA patients and is a hallmark of the condition (24). This could be attributed to increased age, diastolic dysfunction, atrial fibrillation, and coexistent atrial myopathy from atrial amyloid deposition (25). Previous reports have shown that the left atrium (LA) size is smaller in AFD compared with HCM (26), and mild LA dilatation appears to be the characteristic phenotype of AFD.

LV systolic function by LVEF was slightly reduced in CA, whilst LVGLS (a more sensitive marker of LV dysfunction) was markedly reduced. Reduced LVGLS is also a marker of early AFD cardiomyopathy (27). Basal segmental strain in CA was markedly reduced compared with mid and apical strain, with a resultant high RAS, as reported previously (5, 28). Reduction in basal strain has been reported in AFD patients compared with healthy controls (22, 29) and was noted in our group with AFD vs. HHT. However, this was due to a reduction in overall LVGLS in AFD, with RAS being similar in both groups. Moreover, RV function (RVFWS and TAPSE) was also reduced in CA but relatively preserved in AFD (which was similar to HHT). Previous studies have demonstrated that patients with AFD have better RV function compared with those with CA, despite having similar levels of RV “hypertrophy” (30).

### Differentiating cardiomyopathies with a “hypertrophic” phenotype

Using derived echocardiographic parameters, we distinguished infiltrative and storage causes (CA + AFD) from HHT (EFSR and MSR) and CA vs. AFD (RAS and AMYLI), with reasonable sensitivity and specificity. Whilst EFSR was shown to be a strong predictor of CA vs. HCM and hypertensive patients (6), our study demonstrated poor discrimination between CA and AFD (*p* = 0.058).

Both RAS and AMYLI demonstrated good diagnostic performance for CA. Whilst AMYLI was reported as having a good rule-out value, with a value <2.22 excluding CA diagnosis (8), we found reasonable sensitivity (71%) and specificity (83%) for a value ≥7.6 for differentiating CA from AFD. This is due to increased RWT (increased wall thickness with small cavity size) and increased filling pressures (*E*/*e*′), both of which are hallmarks of CA.

The LDA using LVMI, *e*′, LVGLS and basal strain demonstrated good predictive utility for the three groups. These parameters reflect diverse properties (LV structure, systolic and diastolic function) and are easily measured in routine clinical practice. LDA uses simple linear combinations of these measurements that can be calculated and used clinically to classify a new patient into the phenotype group whose centroid is closest (Euclidian distance). These calculations are easily automated and can be trained in machine learning models to improve classification. Other studies have demonstrated the utility of LDA in machine learning algorithms for medical diagnosis (31). Preliminary studies have demonstrated the utility of artificial intelligence in echocardiography for identifying CA and HCM (32, 33), but there are currently no data regarding its utility in AFD except using cardiac magnetic resonance imaging (34). Thus, we can implement our suggested LDA into echocardiographic software for automated screening.

In addition, we demonstrated good accuracy for LDA even in a subgroup of patients with mild–moderately increased wall thickness (MWT <16 mm). Pagourelias et al. (6) demonstrated the accuracy of EFSR in differentiating CA from HCM and HHT in a subgroup with milder “hypertrophy” (wall thickness 12–16 mm). In the subgroup, LDA correctly predicted a similar proportion of cases as that of the entire group (79%), suggesting that this model is robust even in early disease stages.

### Clinical implications

**Figure 5:**
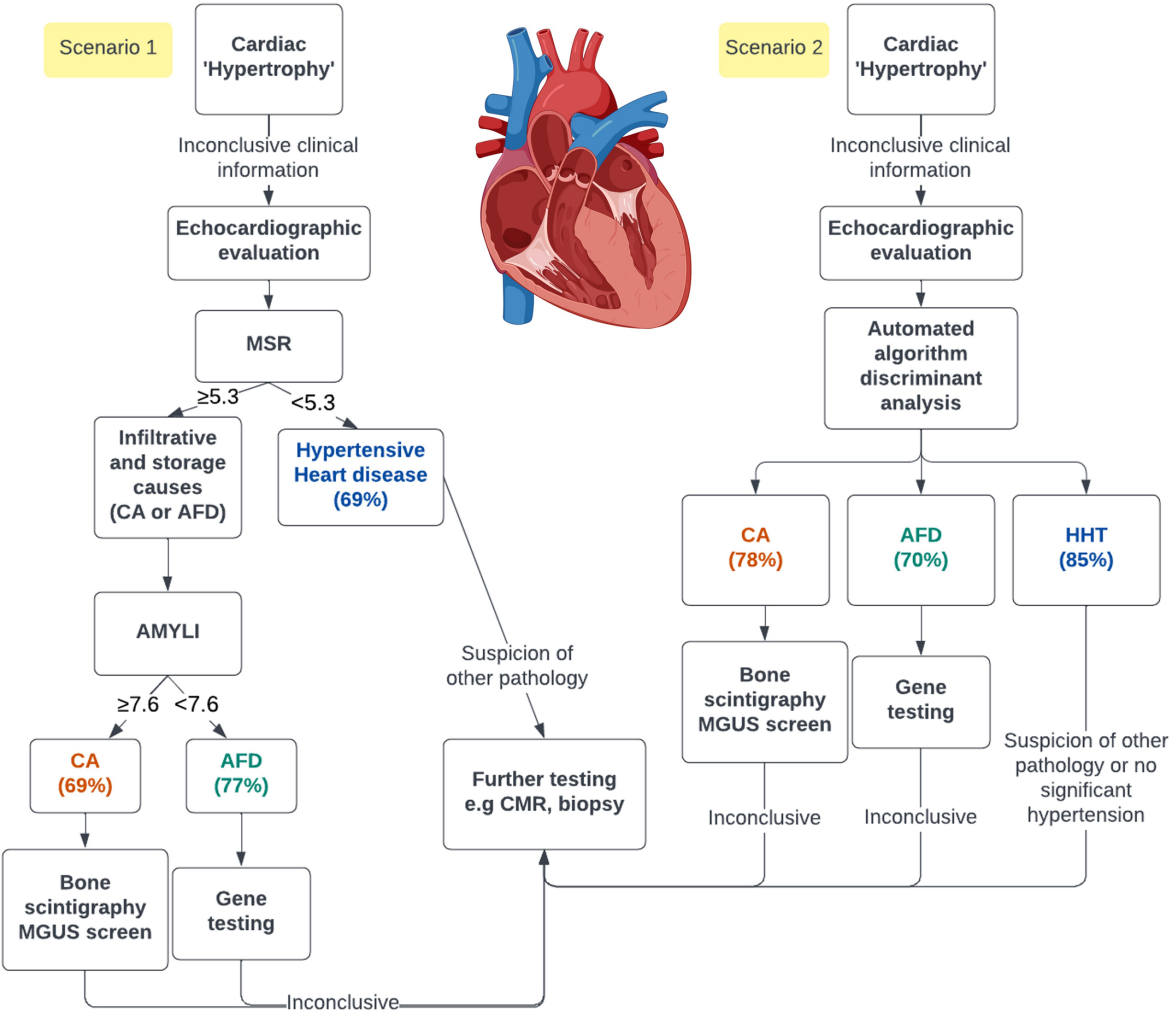
Central illustration. Demonstration of the opportunity of using either simple echocardiographic formulas (scenario 1) or a linear discriminant analysis in an automated algorithm (scenario 2) to screen patients with echocardiographic cardiac “hypertrophy.” The percentage predicted for each group using both methods is represented. AMYLI score, relative wall thickness × *E*/*e*′; MGUS, monoclonal gammopathy of uncertain significance.

Infiltrative and storage aetiologies of cardiac “hypertrophy,” including CA and AFD, are often misdiagnosed or diagnosed late (1, 35), with inevitable treatment delays and increased morbidity. Specialised tests such as CMR or an invasive biopsy can significantly increase healthcare expenditure. Echocardiography, which is inexpensive and widely available, could expedite simple screening, facilitating prompt referral for specific diagnostic testing. As demonstrated, this can be achieved either with simple formulas (e.g., MSR or AMYLI) or with LDA, which can be integrated into echocardiographic software. A proposed clinical algorithm is demonstrated in the *central illustration* (Figure 5). Thus, the benefit of these techniques is in facilitating early diagnosis, whilst also rationalising downstream investigations. Notably, these echocardiographic algorithms should be utilised to complement clinical information (e.g., demographics, clinical history and examination findings and ECG data) and not as definitive diagnoses.

### Study limitations

This study was retrospective in nature, and an independent validation sample of patients from the three phenotypes of interest was not available. Some RV parameters were excluded, especially in the AFD patients with suboptimal RV views. However, we demonstrate that accurate phenotyping is possible utilising only LV parameters. We did not include a comparison with the IWT score (7) as it is a complex score. The proponents of the IWT subsequently developed the simplified AMYLI score evaluated in this study. We did not include HCM for comparison in this study as echocardiographic markers in HCM have been extensively studied before (5–7), and this study focused on CA and AFD. HHT patients had amyloidosis excluded based on clinical criteria and did not have bone scintigraphy. Thus, occult amyloidosis could not be excluded, although we feel this is unlikely as there remained good sensitivity differentiating HHT and secondary aetiologies with the above algorithms.

More specific echocardiographic measurements, including LA strain and regional strain (e.g., LV posterolateral strain), which have shown some utility in CA (36) and AFD (37), were not explored in this study as they are complex to perform, and we sought to incorporate simple measurements and formulae for clinical utility. Another inherent limitation is the vendor dependency of strain analysis, particularly in the case of GE EchoPAC (used in this study), which is vendor-specific. However, recent studies have demonstrated good agreement between vendor-independent and vendor-specific software for LV global longitudinal strain (38). Whilst electrocardiogram data (e.g., low voltage) are useful in these conditions, their use in screening tools is limited by low sensitivity (39). Thus, this was not analysed for inclusion in our algorithms. Moreover, the benefit of echocardiographic algorithms is that they can be potentially incorporated directly into echocardiographic software for automated screening. Finally, we did not incorporate biochemical markers (troponin, NT-proBNP) because the assays differed across time points and sites, reflecting real-life practice.

## Conclusion

Simple echocardiographic measurements differentiate cardiac “hypertrophic” phenotypes, namely, amyloid, Anderson–Fabry and hypertensive heart diseases. We demonstrate that LDA, comprising simple parameters, is effective in accurately differentiating groups. The findings suggest that LDA or other supervised learning classifiers have the potential to be used in automated echocardiographic machine learning algorithms as screening tools. Such an approach would facilitate the early identification of “hypertrophic” phenotypes, guiding downstream confirmatory testing.

## Data Availability

The raw data supporting the conclusions of this article will be made available by the authors, without undue reservation.
