# Supplementary material for: Cardiac “hypertrophy” phenotyping: differentiating aetiologies with increased left ventricular wall thickness on echocardiography
Source: Front Cardiovasc Med. 2023 Jul 3;10:1183485. doi: 10.3389/fcvm.2023.1183485 (PMC10351962; doi:10.3389/fcvm.2023.1183485)
Supplement: Supplementary file 1 [file Datasheet1.docx]

**SUPPLEMENTARY DATA**

**Canonical Discriminant Functions For Linear Discriminant Analysis (figure 4)**

**Discriminant function 1: (1.153*Basal Strain) – (0.164*Average e’) – (0.294*GLS) – (0.001*LVMI)**

**Discriminant function 2: (0.950*Average e’) + (0.616*GLS) + (0.826*LVMI) – (0.346*Basal Strain)**

**Age-adjustment of parameter ratios**

Secondary infiltrative/storage causes (CA + AFD) versus HHT

| **Age-Adjusted** | **Mean Difference** | **95% CI** | **P-value** |
| --- | --- | --- | --- |
| **EFSR** | 1.022 | 0.714-1.331 | <0.001 |
| **MSR** | 6.661 | 5.100-8.222 | <0.001 |

CA vs AFD

| **Age-Adjusted** | **Mean Difference** | **95% CI** | **P-value** |
| --- | --- | --- | --- |
| **RAS** | 0.158 | 0.021-0.294 | 0.024 |
| **AMYLI** | 4.383 | 0.983-7.783 | 0.012 |

*AFD: Anderson-Fabry disease, AMYLI score: RWT x E/e’, BMI: Body mass index, CA: Cardiac amyloid, CI: confidence interval, EFSR: LVEF to strain ratio, HHT: hypertensive heart disease, MSR: LV mass-to-strain ratio, RAS: relative apical sparing.*

**Subgroup comparison MWT<16mm**

Differences in echocardiographic formulas between CA, AFD and HHT in subgroup of patients with mean wall thickness <16mm.

|  | **CA (n=72)** | **AFD (n=24)** | **HHT (n=58)** | **Total (n=209)** | **p-value*** |
| --- | --- | --- | --- | --- | --- |
| EFSR | 3.88 (3.23-4.68) | 3.42 (3.22-4.2) | 3.24 (3.05-3.48) | 3.44 (3.14-4.05) | <0.001 |
| MSR | 8.28 (6.22-11.52) | 7.37 (5.75-9.06) | 4.95 (4.31-5.56) | 6.1 (4.9-8.82) | <0.001 |
| AMYLI | 8.12 (5.67-10.01) | 5.15 (4-6.41) | 5.91 (5.03-7.71) | 6.44 (5.09-8.86) | <0.001 |
| RAS | 0.767 (0.667-0.921) | 0.61 (0.535-0.734) | 0.645 (0.561-0.709) | 0.692 (0.593-0.812) | <0.001 |

**p-value derived from Kruskal-Wallis nonparametric analysis of variance. Data presented as median (lower quartile-upper quartile) for continuous variables. AFD: Anderson-Fabry disease, AMYLI score: RWT x E/e’, BMI: Body mass index, CA: Cardiac amyloid, EFSR: LV ejection fraction to strain ratio, HHT: hypertensive heart disease, MSR: LV mass-to-strain ratio, RAS: relative apical sparing.*


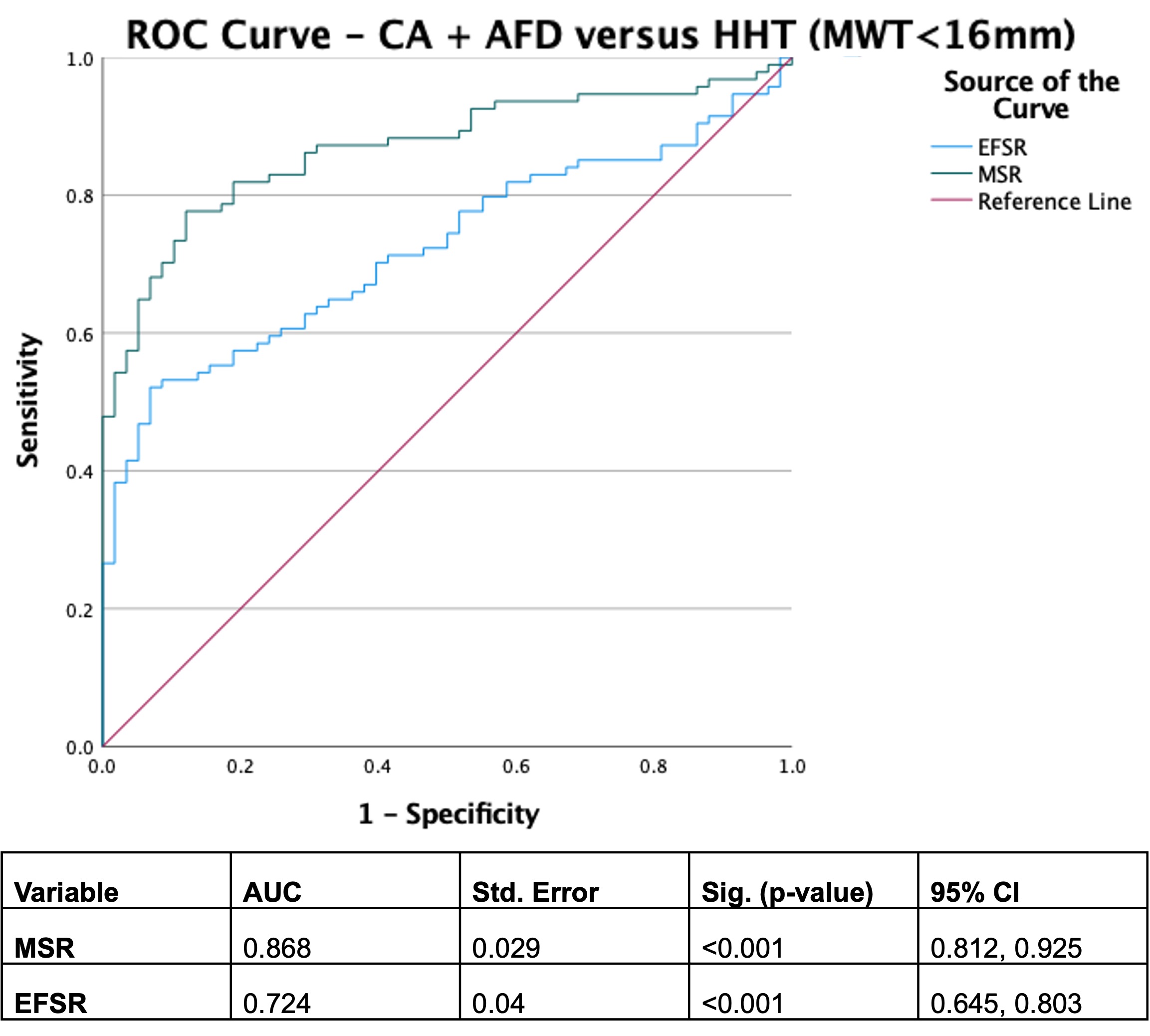


**Supplementary Figure 1.** Receiver operating characteristic (ROC) curve for secondary causes (Cardiac Amyloid and Anderson Fabry Disease) vs hypertensive cardiomyopathy in subgroup of patients with mean wall thickness<16 mm. *AFD: Anderson-Fabry disease, CA: Cardiac amyloid, EFSR: LV ejection fraction to strain ratio, HHT: hypertensive heart disease, MWT: Mean wall thickness, MSR: LV mass-to-strain ratio.*


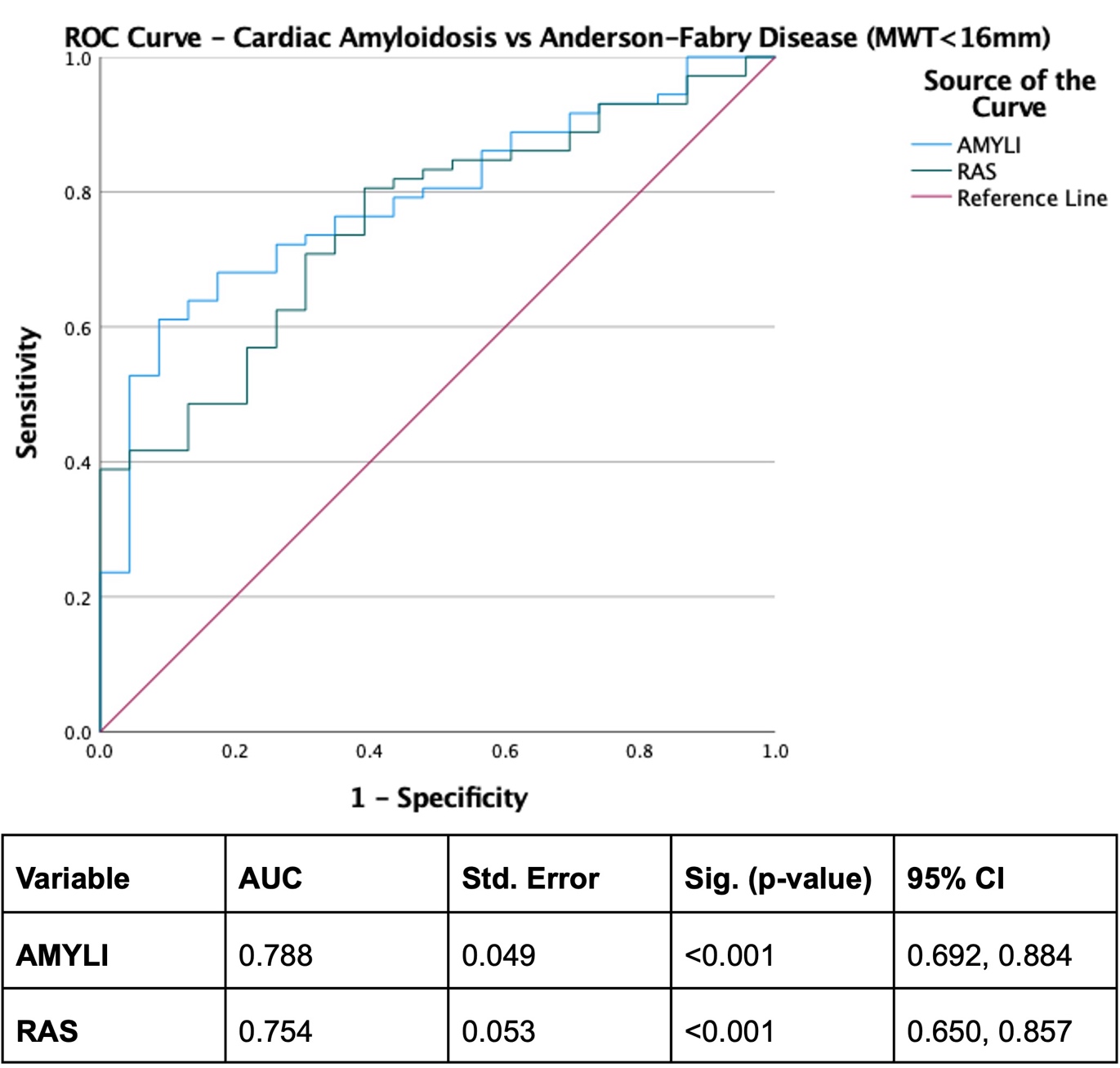


**Supplementary Figure 2.** Receiver operating characteristic (ROC) curve for differentiating Cardiac amyloidosis from Anderson-Fabry disease in subgroup of patients with mean wall thickness <16mm. *AMYLI score: Relative wall thickness x E/e’, MWT: Mean wall thickness, RAS: relative apical sparing.*


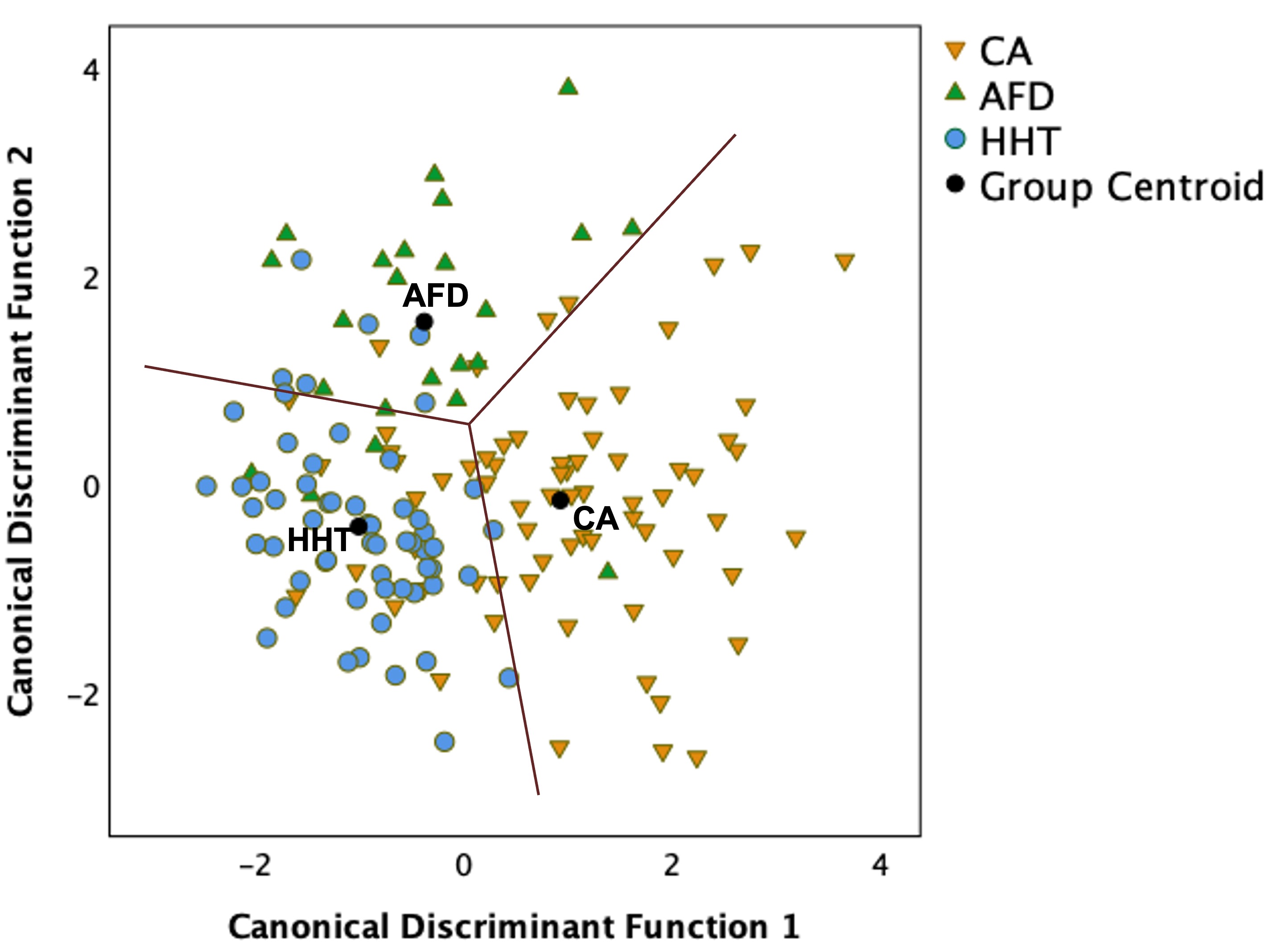


**Supplementary Figure 3.** Scatterplot of the two canonical discriminant functions (linear combinations of Left ventricular mass index, average e’, and basal strain) derived using linear discriminant analysis with stepwise variable selection to differentiate between the 3 groups in subgroup with mean wall thickness <16 mm. Overall 78.9% of cases correctly classified using leave-one-out cross-validation, comprising 75% cardiac amyloidosis (CA), 83% Anderson-Fabry (AFD) and 83% hypertensive heart disease (HHT).
